# Time-varying caloric vestibular stimulation for the treatment of neurodegenerative disease

**DOI:** 10.3389/fnagi.2022.1049637

**Published:** 2022-11-09

**Authors:** Robert D. Black, Eduardo Chaparro

**Affiliations:** ^1^Scion NeuroStim, Durham, NC, United States; ^2^Department of Neurosurgery, Duke University, Durham, NC, United States

**Keywords:** neurodegenerative disease, cerebral blood flow, sensory neuromodulation, time-varying caloric vestibular stimulation, Parkinson’s disease

## Abstract

Time-varying caloric vestibular stimulation (tvCVS) is a new form of non-invasive neuromodulation similar to, but different from, diagnostic caloric vestibular stimulation (CVS). Using a non-invasive, solid-state delivery device, tvCVS has been successfully used in a human clinical trial with Parkinson’s disease (PD) subjects. Additionally, the effects of tvCVS on brain activation have been studied in healthy human subjects using transcranial Doppler sonography (TCD) and functional magnetic resonance imaging (BOLD fMRI). A novel finding in the TCD and fMRI studies was the induction of cerebral blood flow velocity (CBFv) oscillations. How such oscillations might lead to the observed clinical effects seen in PD subjects will be discussed. Enabling studies of tvCVS with rodents is an attractive goal in support of explorations of the mechanism of action. Male Wistar rats were used in a proof-of-concept study described herein. Rats were anesthetized (isoflurane) and ventilated for the duration of the tvCVS runs. Time-varying thermal stimuli were administered using a digital temperature controller to modulate Peltier-type heater/cooler devices. Blunt ear bars conveyed the thermal stimulus to the external ear canals of the rats. Different thermal waveform combinations were evaluated for evidence of successful induction of the CVS effect. It was found that bilateral triangular thermal waveforms could induce oscillations in CBFv both during and after the application of tvCVS. These oscillations were similar to, but different from those observed in awake human subjects. The establishment of a viable animal model for the study of tvCVS will augment ongoing clinical investigations of this new form of neuromodulation in patients with neurodegenerative disease.

## Introduction

Caloric vestibular stimulation (CVS) has been used in diagnostics for decades as a tool to study balance disorders. The diagnostic CVS units use water or air as the working fluid to irrigate the external ear canal at temperatures above and below body temperature (Fitzgerald and Hallpike, 1942). Ear canal irrigation leads to the induction of thermoconvection currents in the vestibular organs, most particularly the horizontal semicircular canal, where there is a concomitant change in the afferent firing rate of the vestibular hair cells, which is communicated to the vestibular nuclei in the brainstem. The broad extent of direct and indirect innervation of brain regions by the vestibular sensory network makes CVS an attractive candidate for therapeutic neuromodulation (Black and Rogers, 2020). Water-based (irrigation) CVS has been used in previous clinical studies to explore therapeutic effects. McGeoch et al. (2009) and Ramachandran et al. (2007) provided evidence of transient pain reduction after a single CVS session. Holé et al. (2020) summarize the substantial literature examining the transient reduction in hemispatial neglect resulting from acute CVS treatment. Time-varying caloric vestibular stimulation (tvCVS) is a different form of non-invasive neuromodulation that has been evaluated in clinical studies with human subjects (Vanzan et al., 2017; Wilkinson et al., 2017, 2019). Early evidence suggests that tvCVS can induce oscillations in cerebral blood flow as well as alter regional neurovascular coupling (Black et al., 2016, 2021).

Here, we describe a tvCVS device adapted to a stereotactic frame where blunt ear bars were modified to convey a contact-based thermal stimulus in rodents. The temperature of the ear bars was regulated by a closed-loop control circuit driving solid-state heater/cooler elements (Peltier devices). Preliminary, proof-of-concept testing provided evidence that the tvCVS system successfully delivered thermal energy to the vestibular labyrinth. In particular, cerebral blood flow velocity (CBFv) oscillations, as measured with laser Doppler flowmetry, in response to a time-varying CVS stimulus pattern were observed. The demonstration of similar flow oscillations induced in human subjects facilitates a novel platform to study changes in brain-blood-flow associated with a range of disease processes (including neurodegenerative diseases).

## Materials and methods

The Duke University Institutional Animal Care and Use Committee approved the use of the tvCVS neuromodulation device under the experimental conditions listed below. Male Wistar rats (12 weeks of age) were fasted from food for 8–12 h to deplete liver glycogen thereby controlling plasma glucose concentration. Rats were anesthetized with isoflurane in 50% O_2_/balance N_2_ in an induction box. Following tracheal intubation, the lungs were mechanically ventilated to maintain normocapnia (anesthesia and monitoring were coordinated using the MacLab 8e system, ADInstruments, Sydney, NSW, Australia). Aseptic technique was used. A tail artery catheter was placed for blood pressure monitoring. The wound was infiltrated with 0.25% bupivacaine. A 1-cm scalp incision was made, and a burr hole was drilled through the outer table of the skull (inner table left intact) 1-mm posterior to bregma and a 6-mm right lateral of midline. A laser Doppler flow probe (Moor Instruments, Wilmington, DE, United States) was temporarily placed at this site to evaluate red blood cell flow velocity as a surrogate measure for cerebral blood flow. Laser Doppler flowmetry is a convenient tool for studying real-time flow changes (Taninishi et al., 2015). A pericranial thermistor was percutaneously implanted beneath the right termporalis muscle allowing servo-regulated control of temperature within 37.5 ± 0.1°C using surface heating/cooling as required. Inspired isoflurane concentration was stabilized at 1.2 ± 0.2%. At the end of the study, isoflurane exposure was increased to 5% and held until respiration ceased and death ensued as approved by protocol. Additional information on the methodology described above can be found in Hoffmann et al. (2016) and Pittman et al. (1997).

**FIGURE 1:**
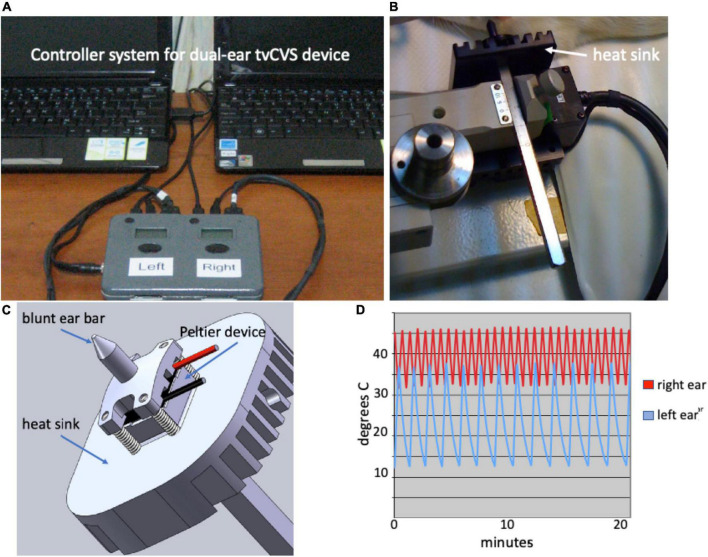
**(A)** Dual ear Time-varying caloric vestibular stimulation (tvCVS) system; **(B)** Caloric ear bar and heat sink; **(C)** Schematic of caloric ear bar; **(D)** Example thermal waveforms.

A tvCVS unit that employs Peltier devices to warm/cool the ear canals was designed and constructed so that it would fit with a stereotactic positioning system. Figure 1A shows the control system, allowing for independent thermal waveforms to be delivered to both ears (Figure 1D). Temperature sensors mounted near the ear bar tips provided feedback to a temperature control algorithm (proportional-integral-derivative control loop) and the system was calibrated using an independent thermocouple-based meter. A Peltier device was mounted between an aluminum heat sink and an anodized aluminum, blunt ear bar tip (Figure 1C). Those structures were then affixed to a stainless steel, graduated shaft designed to fit into an adjustable clamp on the stereotactic rig (Figure 1B).

The triangular thermal waveforms were chosen based on the thermal output capabilities of the caloric ear bar device and to avoid adaptation of vestibular hair cells. The use of a constant temperature for CVS will result in reduced responsiveness of the hair cells after several minutes (Bock et al., 1979) and thus by continuously varying the temperature adaptation effects are frustrated. Figure 1D shows a combination of a warm waveform (varying from 33 to 46) and a cold waveform (varying from 13 to 37). The upper temperature limit reflects that of diagnostic CVS and the lower limit is a function of the capability of the cooling system.

**FIGURE 2:**
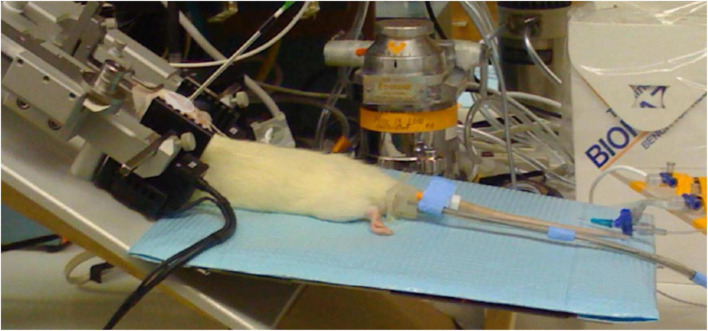
Caloric ear bars in place, laser Doppler probe affixed; head flexed upward by 30 degrees, support pad tilted slightly upward.

For tvCVS stimulation the rat was placed in a stereotactic head frame and the caloric ear bars were placed in the ear canals without damaging the tympanic membrane (Figure 2). The total duration of the tvCVS stimulus did not exceed 60 min. The thermal stimulus was applied to one or both ears simultaneously. The horizontal semicircular canal, the structure that dominates induction of the CVS effect, in rats is tipped (higher anteriorly) by about 30°. CVS is maximal when the orientation of the canal is 90° with respect to the horizontal plane (Fitzgerald and Hallpike, 1942). Therefore, the rat was studied with a combination of a tilt of the body axis and upward flexion of the head to improve the likelihood of strong CVS induction. It was not practical to achieve a 90° orientation of the horizontal canal (corresponding to a 60° tilt of the body axis), but the configuration shown in Figure 2 proved to enable good induction of the CVS effect as discussed below.

## Results

The method for delivering tvCVS was found to be effective and took advantage of good familiarity with stereotactic fixation protocols. Ensuring good registration of the ear bars was straightforward. Accommodating the need to tilt the rat so that the horizontal semicircular canal was closer to a vertical orientation required a number of tests. Initially, tilting the animal platform to about 50° above the horizontal was evaluated. This proved to be cumbersome and the platform was repositioned to be horizontal. A number of runs were completed in the horizontal orientation before another modification was evaluated with the rat’s body tilted slightly above horizontal and with an upward head flexion of about 30° (Figure 2). This proved to be an improvement in terms of modulating CBFv, a key observation in this study. The maintenance of anesthesia was antagonized by the known arousal effect of CVS (e.g., Ferrè et al., 2011). The animals needed constant monitoring of anesthetic status to ensure complete anesthesia during the procedure.

Two different thermal waveform delivery approaches are described here: (1) single ear, cold triangular and (2) dual ear, warm, and cold triangular waves (the combination shown in Figure 1D). Control runs (ear bars in place, no temperature change) were executed periodically. Waveform configurations 1 and 2 were noteworthy as they resulted in the induction of flow velocity oscillations as measured with the Doppler flowmeter (Figure 2). The Doppler flow time series data were analyzed using a fast Fourier transform algorithm (StatPlus, AnalystSoft Inc., Alexandria, VA, United States) in order to view the spectral power data.

**FIGURE 3:**
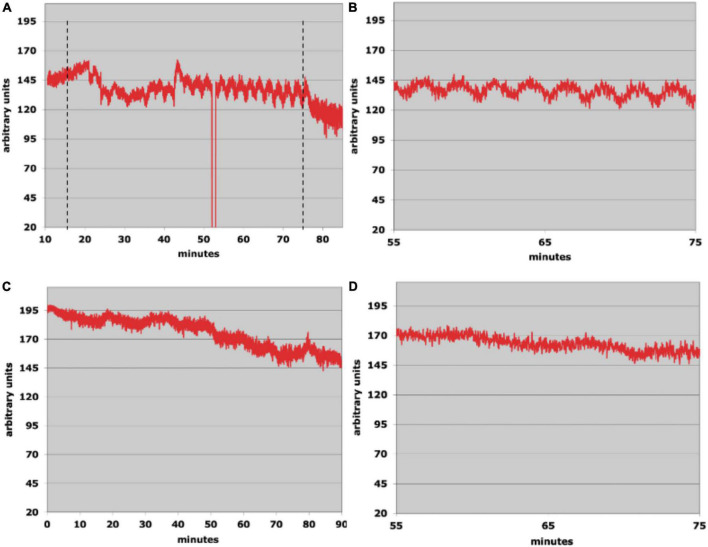
**(A)** Laser Doppler flowmetry data; the dotted lines show the start and stop times of the time-varying caloric vestibular stimulation (tvCVS) stimulation (the glitch at ∼53 min was due to the recording system falling out briefly and did not affect the measurement otherwise). **(B)** An expanded view of the oscillations from panel **(A)**. **(C)** A control run (no tvCVS stimulation). **(D)** An expanded view of a segment of the control run.

Figures 3A,B show the results from a run using waveform type 1 with the rat in a horizontal position (no head flexion). A triangular waveform alternating between 18 and 37°C with a period of 1.8 min was applied to the right ear. Horizontal markers show the start and stop times for the tvCVS stimulation. After about 5 min into the run, the flow signal starts to alter showing some evidence of structure. By the 45-min mark (30 min after tvCVS start), clear oscillations begin to emerge. Figure 3B shows an expanded x-axis. The periods of the induced oscillations vary in a range of ∼2.0–2.5 min. Interestingly, this is slower than the period of the applied tvCVS thermal waveform. Once the stimulation stopped (∼75-min mark), the oscillations quickly damped out. A control run was conducted the following day and is shown in Figures 3C,D. There is no corresponding evidence of induced oscillations in the control run.

**FIGURE 4:**
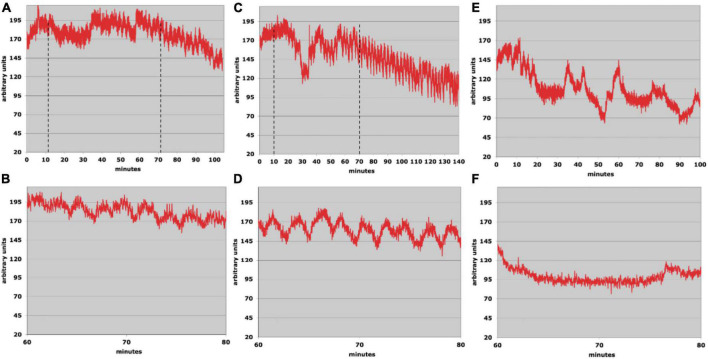
**(A,C)** Laser Doppler flowmetry data; the dotted lines show the start and stop times of the time-varying caloric vestibular stimulation (tvCVS) stimulation. **(B,D)** Expanded views of the oscillations from panels **(A,C)**. **(E,F)** Control run.

Figure 4 shows results from two runs using waveform type 2. The rat was positioned as shown in Figure 2. A warm waveform (varying from 33 to 46°C) was administered to the right ear and a cold waveform (varying from 13 to 37°C) was administered to the left. The period of the warm waveform was 0.65 min and that of the cold was 1.7 min. In both of the active runs, the flow oscillations seen previously emerged again, however, they persisted past the end of the stimulation period without abatement until the experimental session was finally terminated. In Figures 4C,D, the time of the run after tvCVS stimulation was extended to see if the oscillations would eventually stop, but they continued until it was necessary to end the session due to time constraints.

The oscillation period in the run of Figures 4A,B varied from about 2.0 to 2.5 min. As before, the oscillation period was slower than the periods of the applied thermal waveforms. For the run of Figures 4C,D, the oscillations were stronger and also ranged from 2.0 to 2.5 min. The range of oscillation periods overlapped those found in the single-ear run (Figure 3), suggesting that an endogenous period was entrained by the tvCVS stimulus. The control run (Figures 4E,F) showed no evidence of flow oscillations, however, the Doppler probe signal intensity shifted several times (most likely due to the probe contact not being entirely stable).

## Discussion

**FIGURE 5:**
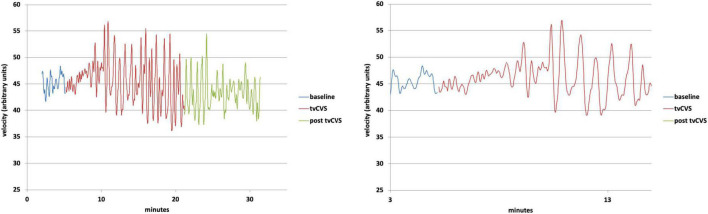
**Top:** Doppler ultrasound mean velocity (using 10 nearest-neighbor averaging); **Bottom:** Expanded view of the transition from baseline to time-varying caloric vestibular stimulation (tvCVS) stimulation.

It should be emphasized that the preliminary results reported above are exploratory and were acquired while evaluating the overall operation of the tvCVS rig. Significant additional work will be required to use this procedure and understand how to consistently induce the flow effect being reported. The aim of this study was to develop a methodology for delivering tvCVS to anesthetized rats in support of future animal research. The observation of CBFv oscillations, dependent on the type of thermal waveform used, was a novel finding and became the focus of investigations with human subjects. Previous CVS studies in humans (Tiecks et al., 1996; Heckmann et al., 1999) demonstrated monotonic changes (increase/decrease) in CBFv and so it is not too surprising that an oscillating tvCVS waveform led to oscillations in CBFv. The induction of oscillations in autonomic parameters using other forms of vestibular stimulation has also been reported. Cohen et al. (2011) used sinusoidal galvanic vestibular stimulation (sGVS) in a frequency range of 0.025–0.5 Hz to measure changes in blood pressure (BP) and heart rate (HR) in rats. They found a sinusoidal variation in BP and HR matched to an integral multiple of the GVS frequency. Serrador et al. (2009) used a high-torque hydraulic-powered tilt chair to vary the orientation of a human subject over a frequency range of 0.03125–0.5 Hz. The authors measured oscillations in cerebral flow velocity in the middle cerebral artery and in BP. Neither of these two previous studies showed evidence of entrainment past the end of the stimulus period. For comparison, in the results reported here the thermal waveform frequencies used for rats varied from 0.01 to 0.025 Hz and those for humans were between 0.008 and 0.017 Hz (see Figure 5). B waves are generally defined to be in a range from 0.008 to 0.05 Hz (Spiegelberg et al., 2016).

The periods of the applied thermal waveforms did not exactly match the period of the flow oscillations. We hypothesize that the time-varying CVS stimulus entrained an endogenous rhythm, possibly associated with B waves (Spiegelberg et al., 2016). Fluctuations in vessel diameters associated with B waves may be due to monoaminergic and serotonergic centers in the midbrain and pons (Sliwka et al., 2001). B waves are believed to be important for autoregulation (Lemaire et al., 2002) and are present unless brainstem death has occurred (Lescot et al., 2005). We hypothesize that once the stimulation was no longer driving the flow oscillations, the natural, entrained B wave period emerged. That the flow oscillations persisted well after the end of tvCVS is perhaps consistent with the changes in effect under anesthesia, since isoflurane can interfere with autoregulation (Strebel et al., 1995; Dagal and Lam, 2009) and therefore may have altered B wave dynamics.

Chronologically, this work was followed by studies with human subjects, using a warm/cold thermal waveform combination. Figure 5 shows results (using the dataset from Black et al., 2016) from a human subject with a warm/cold applied thermal waveform pair: left-ear cold, 17–37°C (2-min period); right-ear warm, 37–42°C (1-min period). Note the nearly immediate oscillations in flow velocity, but not resonance (increased amplitude) until around the 4-min mark. The change in flow velocity once resonance occurred was a 20–30% effect, versus the 4–6% change reported previously for constant temperature CVS (Tiecks et al., 1996; Heckmann et al., 1999). We have proposed that the cyclic application used in tvCVS enables entrainment and thereby amplifies the modulation in flow velocity. The oscillations in flow velocity continue past the end of tvCVS stimulation in Figure 5, but they start to abate after ∼4–5 min, similar to the time needed to induce them. This is in contrast to the sustained oscillations seen in anesthetized rats, again presumably due to the effect of anesthesia on cerebral autoregulation. The period of the oscillations was ∼40 s (1.5 cycles per minute), yet again not precisely matched to the applied thermal waveform periods. This is not surprising since oscillators respond to off-resonance forcing. Performing a Fourier transform of the data in Figure 5 show that there was a narrowing of the spectral peak at 40 s in the post-stimulation period, suggesting that it does represent an endogenous value. This value lies within the range of B wave periods reported in the literature, bolstering the hypothesis that the oscillations arise from the entrainment of a B wave generator.

It is interesting to compare the size of the modulation seen in cerebral blood flow in Figure 5 with other study reports. Tiecks et al. (1996) and Heckmann et al. (1999) saw ∼4–6% modulation effect with a constant temperature water-irrigation based CVS approach. Nishijima et al. (2016) reported on changes in flow velocity in the middle cerebral artery during exercise. The largest change was ∼30%, occurring at 60% of maximal exercise intensity, comparable to the size effect seen in Figure 5. We are unaware of other therapeutic approaches that enable the modulation of CBFv in this way. We hypothesize that these oscillations may be important in underpinning the mechanism of action of tvCVS (Black and Rogers, 2020) perhaps by improving the health and function of the neurovascular unit (Iadecola, 2017). The potential impact of B ways on waste clearance in the brain has been highlighted recently (Fultz et al., 2019; Newell et al., 2022). Indirect evidence of improved neurovascular unit health may have been manifested in the clinical improvements resulting from the use of tvCVS by subjects with episodic migraine (Wilkinson et al., 2017) and with Parkinson’s disease (PD) (Wilkinson et al., 2019). That tvCVS seems efficacious for these two diseases, which are generally not thought to have common etiologies, points to a central mechanism as the means of action. The study with PD patients, in particular, suggested that the effects of tvCVS were broad (improving both motor and non-motor symptoms) and durable. The durability seen in the active treatment group extended to 1 month after the end of treatment without reduction and even at 6 months post-treatment the active group improvements were still above baseline. The hypothesized changes discussed above, such as the improvement of the health and function of the neurovascular unit, would fall within the boundaries on putative mechanisms of action suggested by the PD study.

The work presented here must be viewed as a preliminary, proof-of-concept study of the methodology of delivering tvCVS to rats. Several challenges and limitations should be noted: (1) the study was not statistically powered and instead used a phenomenological approach to power future research; (2) the aforementioned arousing effects of CVS can complicate the maintenance of anesthesia; (3) isoflurane may have altered normal cerebral autoregulation and altered the basal metabolic rate; (4) the low mass of the rat’s skull can lead to temperature changes in the vestibular labyrinth, which must be considered when discussion the details of CVS induction (Stanford et al., 2021); and (5) the use of tvCVS with human subjects, since the clinical device is viewed as a non-significant risk device, is less challenging than with rats, which must be anesthetized and positioned in an atypical orientation. There were no overt side effects noted in human subjects, nor were they stressed by the procedure. Developing a means for studying animal models with the tvCVS approach is an attractive aim since it would enable invasive measurements, for example real-time assays using brain microdialysis methods and the collection of serial (in time) histopathological sections (Nishijima et al., 2010).

## Future directions and potential healthcare impact

Home-use neuromodulation devices are under active development. The device used in previous clinical studies (Black et al., 2016) has been updated to improve ease of use for PD patients and is now being evaluated in a multi-center study (NCT04797611). tvCVS is a novel neuromodulation approach that capitalizes on the extensive vestibular neuroscience knowledge generated by diagnostic CVS studies. The use of the endogenous vestibular sensory network, a form of sensory neuromodulation (Black and Rogers, 2020), provides potential advantages in terms of safety (natural limits on stimulus intensity), specificity (following a defined neuronal network) and extent of anatomic targets [from the brainstem to the neocortex (Black et al., 2021)]. Currently additional fMRI studies are underway to explore the effects of waveform frequency on the strength (BOLD imaging) and extent of activation beyond the brainstem. The development of a rodent-based tvCVS system extends the realm of potential future research studies that are not possible with human subjects.

## Data availability statement

The raw data supporting the conclusions of this article will be made available by the authors, without undue reservation.

## Ethics statement

The animal study was reviewed and approved by Duke University Institutional Animal Care and Use Committee, Durham, NC, USA.

## Author contributions

RB was responsible for the neurostimulation equipment and initial data analysis. EC performed all animal care and handling tasks. Both authors contributed to the writing of the manuscript.
